# Supplementary material for: The Investigation of Giardiasis (Foodborne and Waterborne Diseases) in Buffaloes in Van Region, Türkiye: First Molecular Report of Giardia duodenalis Assemblage B from Buffaloes
Source: Pathogens. 2023 Jan 8;12(1):106. doi: 10.3390/pathogens12010106 (PMC9863494; doi:10.3390/pathogens12010106)
Supplement: Supplementary file 1 [file pathogens-12-00106-s001.zip › pathogens-2079681-supplementary.pdf]

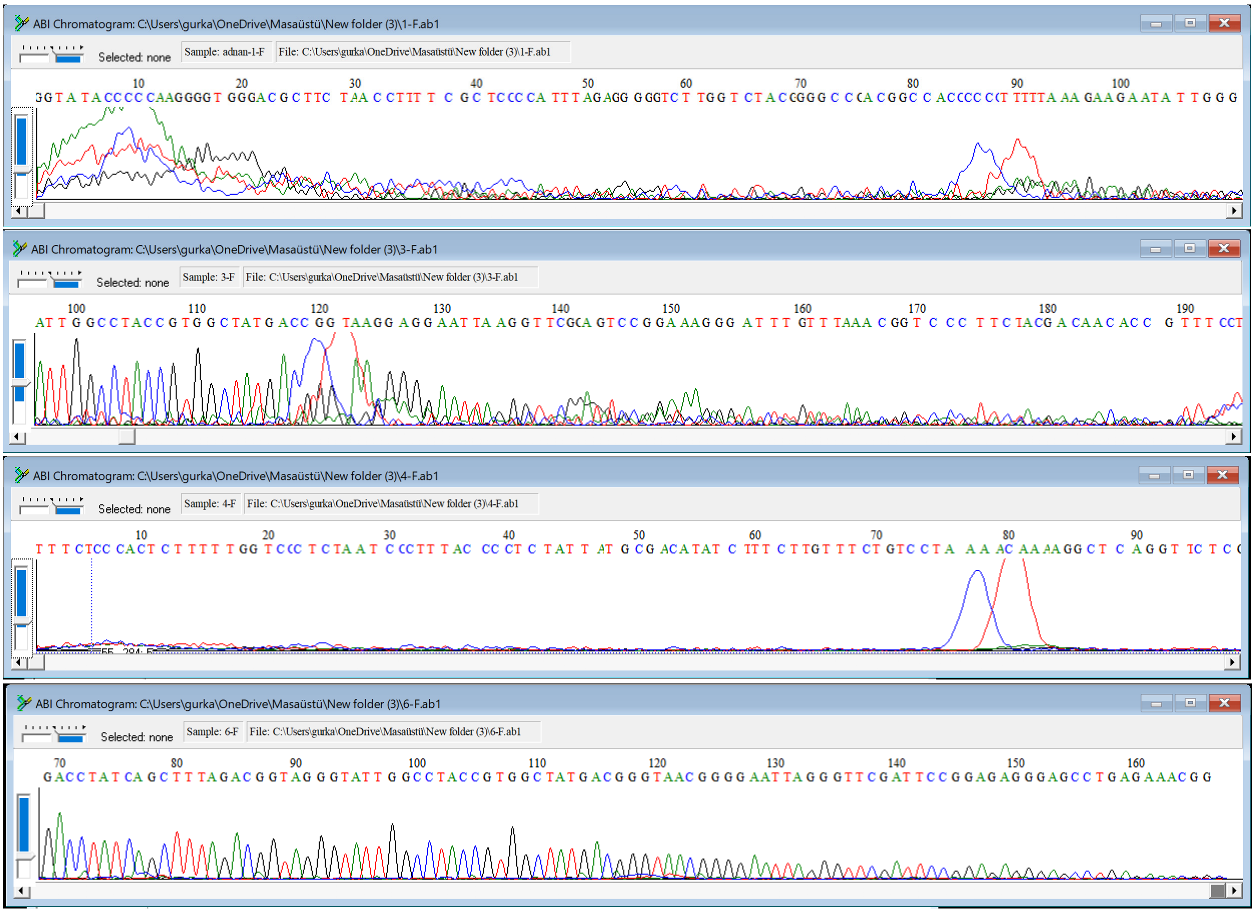

Figure S1. Distorted sequence results All samples were sequenced by sanger dideoxy sequencing; However, it is not given in the article in order not to give an incorrect result due to the early termination of sequencing in some of the 7 positive samples whose results were not given. You can see the sequence graphs of a few examples with no results below and understand why we didn't include them in the article.

Table S1. Model test results used in the interpretation of sequence analyzes.

Model	#Parameters	BIC	AICc	lnL
TN93+G	63	2823,198578	2368,495149	- 1120,849785
TN93+I	63	2825,743868	2371,04044	-1122,12243
TN93+G+I	64	2828,459772	2366,551538	-1118,86531
T92+G	60	2838,870625	2405,78401	- 1142,531022
T92+G+I	61	2843,903501	2403,610882	- 1140,432389
K2+G	59	2844,000435	2418,120223	- 1149,710999
GTR+G	66	2845,330788	2369,014142	- 1118,070675
K2+G+I	60	2851,302285	2418,21567	- 1148,746852
HKY+G	62	2851,347203	2403,84898	- 1139,539169
GTR+I	66	2851,737851	2375,421205	- 1121,274206
GTR+G+I	67	2852,345143	2368,824891	- 1116,962781
HKY+G+I	63	2852,726711	2398,023282	- 1135,613851

JC+G	58	2888,08524	2469,41183	- 1176,368473
K2+I	59	2890,097187	2464,216974	- 1172,759375
T92+I	60	2891,760784	2458,674169	- 1168,976102
JC+G+I	59	2896,119695	2470,239483	- 1175,770629
TN93	62	2908,042506	2460,544283	-1167,88682
HKY+I	62	2911,718464	2464,220241	- 1169,724799
K2	58	2931,145971	2512,472562	- 1197,898839
JC+I	58	2931,510787	2512,837377	- 1198,081246
GTR	65	2932,529356	2463,416716	- 1166,285031
T92	59	2936,019918	2510,139706	- 1195,720741
HKY	61	2959,525923	2519,233304	-1198,2436
JC	57	2971,556836	2560,090627	- 1222,719342
